# Supplementary material for: Disparate co-evolution and prevalence of sulfadoxine and pyrimethamine resistance alleles and haplotypes at dhfr and dhps genes across Africa
Source: Sci Rep. 2025 Apr 17;15:13222. doi: 10.1038/s41598-025-98035-7 (PMC12006366; doi:10.1038/s41598-025-98035-7)
Supplement: Supplementary file 1 — Supplementary Material 1 [file 41598_2025_98035_MOESM1_ESM.pdf]

# Disparate co-evolution and prevalence of sulfadoxine and pyrimethamine resistance alleles and haplotypes at *dhfr* and *dhps* genes across Africa: Supplementary Materials

Nina F. D. White[1], Georgia Whitton[1], Varanya Wasakul[2], Lucas Amenga-Etego[3,7], Antoine Dara[4,7], Voahangy Andrianaranjaka[5,7], Milijaona Randrianarivelojosia[5,7], Olivo Miotto[2,7], Umberto D'Alessandro[6], Abdoulaye Djimdé[4,7], Cristina V. Ariani[1], Richard D. Pearson[1*], Alfred Amambua-Ngwa[6,7*]

1. Genomic Surveillance Unit, Wellcome Sanger Institute, Hinxton, United Kingdom
2. Mahidol-Oxford Tropical Medicine Research Unit, Faculty of Tropical Medicine, Mahidol University, Bangkok, Thailand
3. West African Center for Cell Biology of Infectious Pathogens, University of Ghana, Accra, Ghana
4. Malaria Research and Training Centre, Faculty of Pharmacy, Université des Sciences, des Techniques et des Technologies de Bamako, Bamako, Mali
5. Institut Pasteur de Madagascar, Antananarivo, Madagascar
6. Medical Research Council Unit, The Gambia at London School of Hygiene and Tropical Medicine, Banjul, The Gambia
7. Pathogens Genomic Diversity Network Africa (PDNA), Sotuba, Bamako, Mali

* Corresponding authors. RDP: rp7@sanger.ac.uk, AA-N: Alfred.Ngwa@lshtm.ac.uk

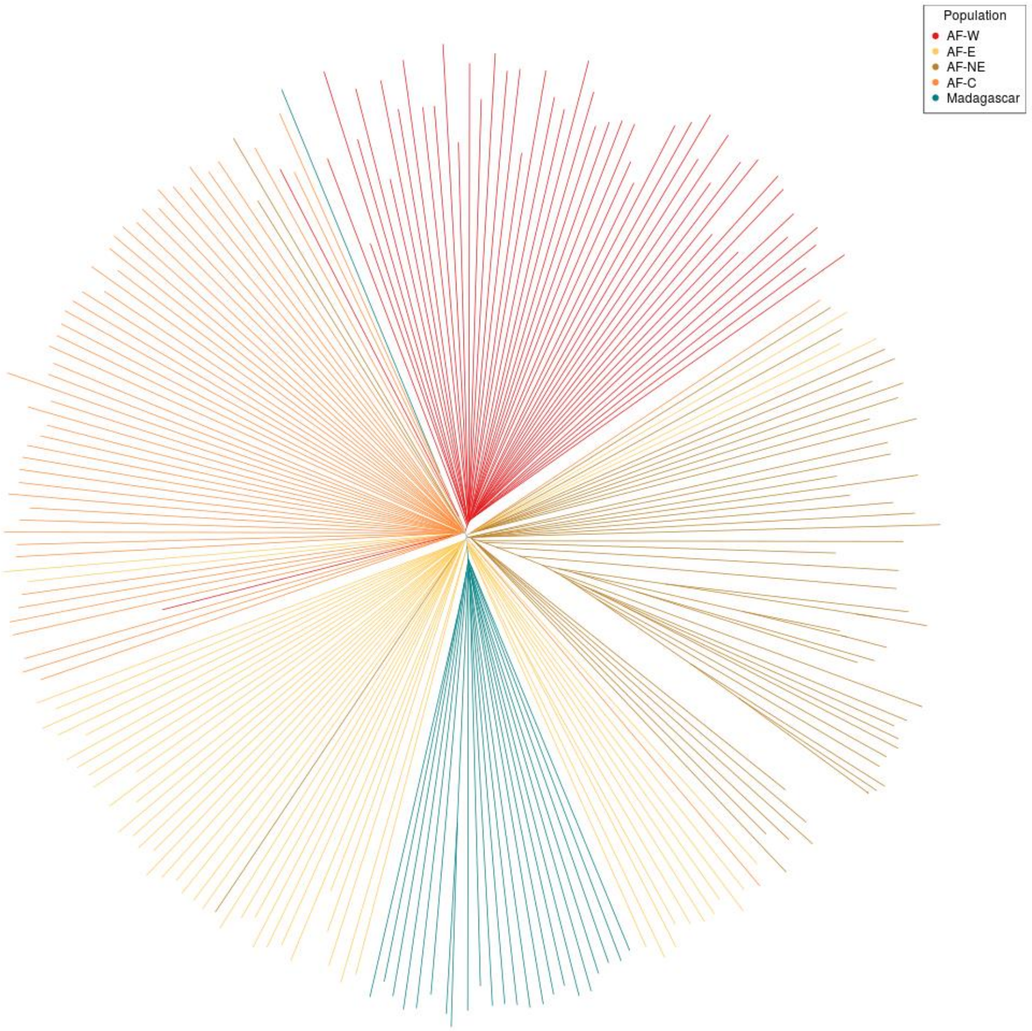


**Supplementary Figure S1**. Genome-wide unrooted neighbour-joining tree between mainland African populations and Madagascar. A random sample of n = 50 QC+ samples was selected for each population: AF-W (west Africa, red), AF-C (central Africa, orange), AF-NE (northeast Africa, brown), and AF-E (east Africa, gold, excluding Madagascar). Madagascar is highlighted in teal and contains n = 24 QC+ samples. Population structure is evident between all populations, but Madagascar is most similar to AF-E.

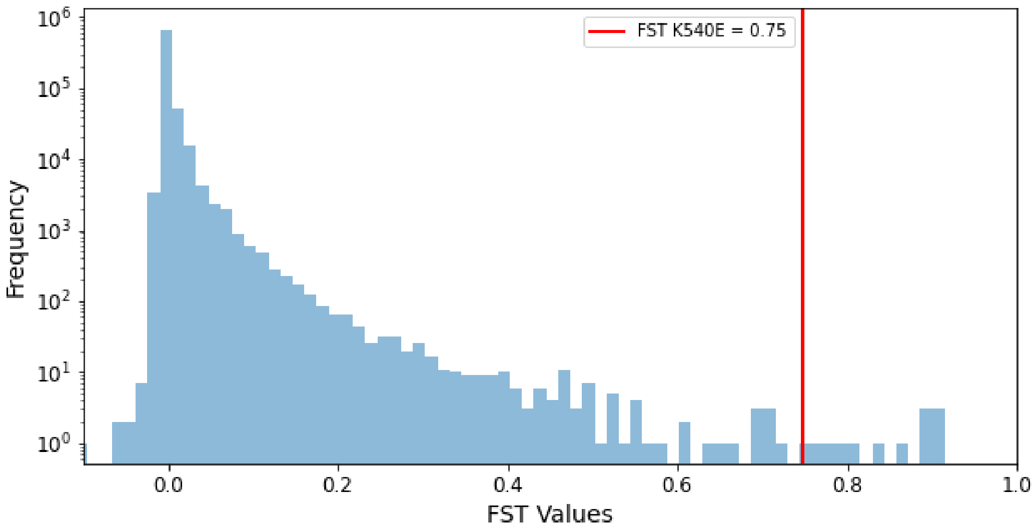


**Supplementary Figure S2**. $F_{ST}$ values for 763,535 genome-wide QC-passed SNPs between Madagascar (n = 24) and the remainder of mainland East Africa (n = 1,515). The FST value for the nucleotide mutation encoding *dhps* K540E is highlighted in red. Frequency is shown on a log scale.

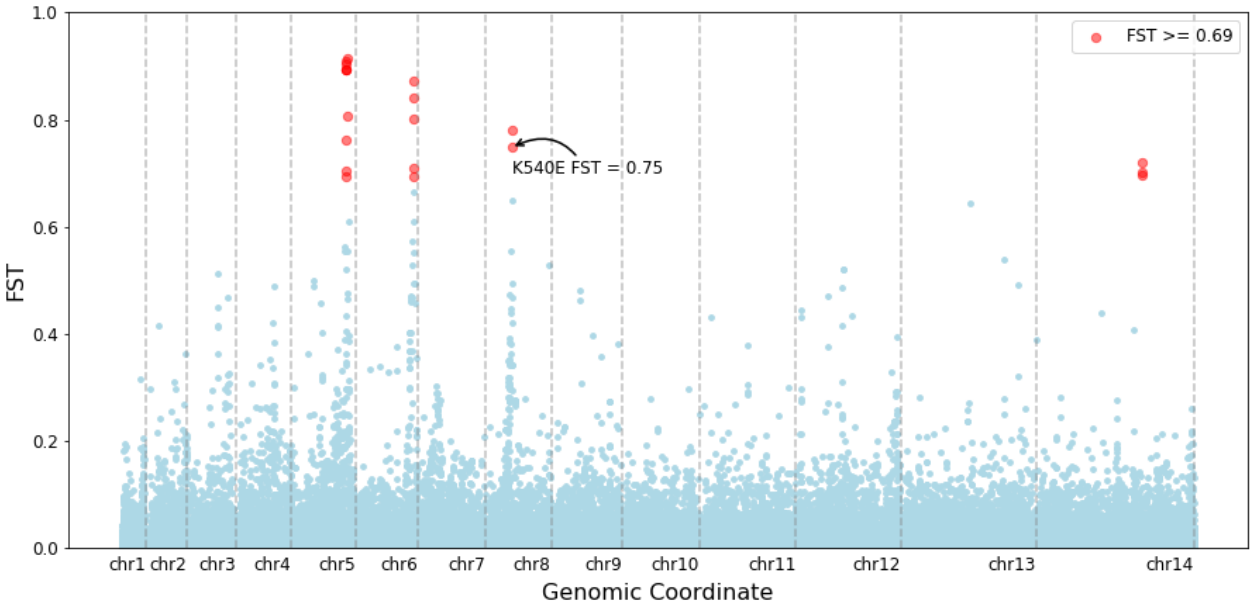


**Supplementary Figure S3**. $F_{ST}$ values for 763,535 genome-wide QC-passed SNPs between Madagascar (n = 24) and the remainder of mainland east Africa (n = 1,515). The 20 most differentiated SNPs ($F_{ST}$ > 0.69) are highlighted, including the position encoding *dhps* K540E.

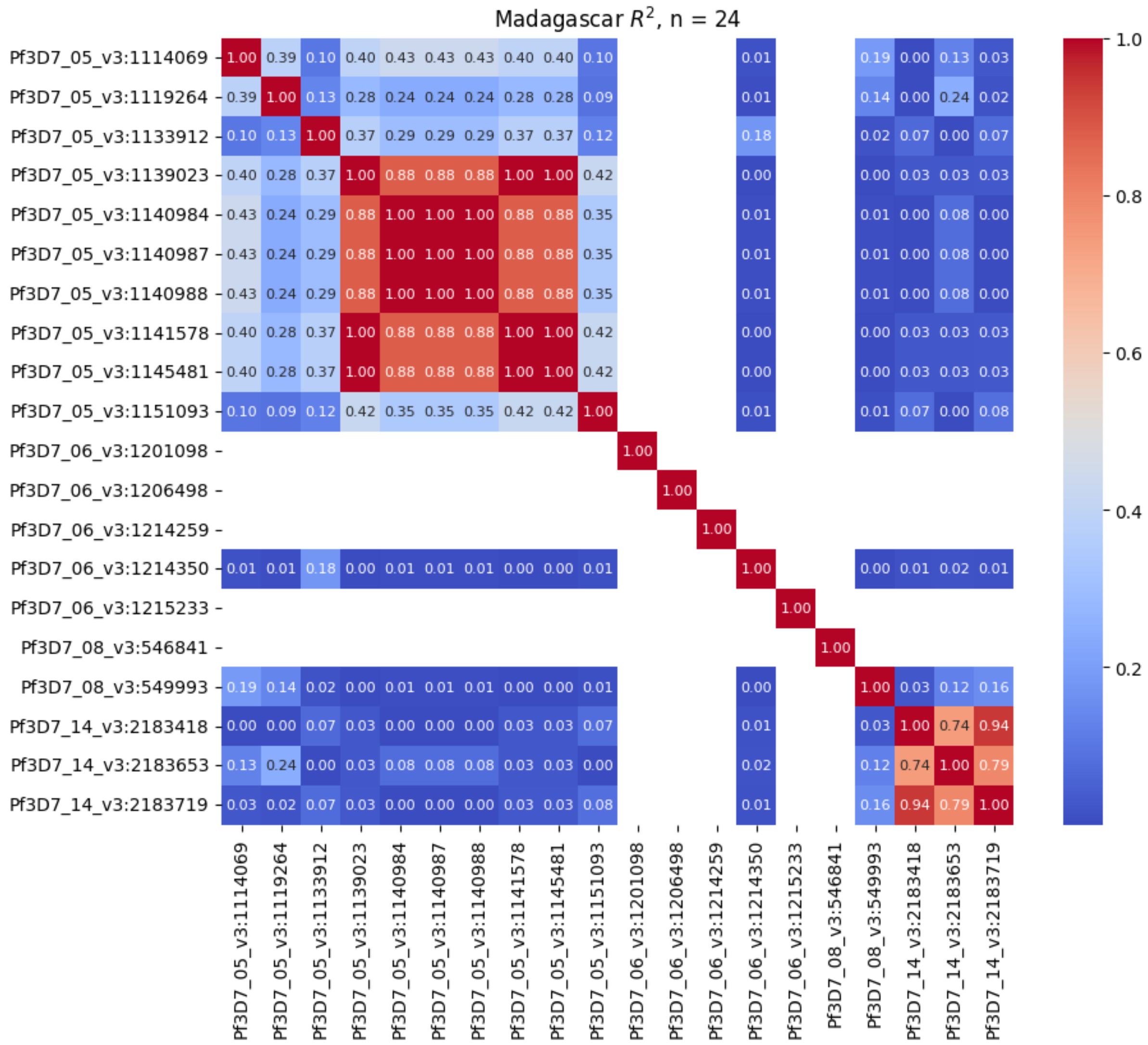


**Supplementary Figure S4**. Linkage disequilibrium ($R^2$) for the 20 most differentiated SNPs between Madagascar and East Africa. Shown here are the $R^2$ values for the Madagascar population. Missing values indicate the SNP was not present in the population.

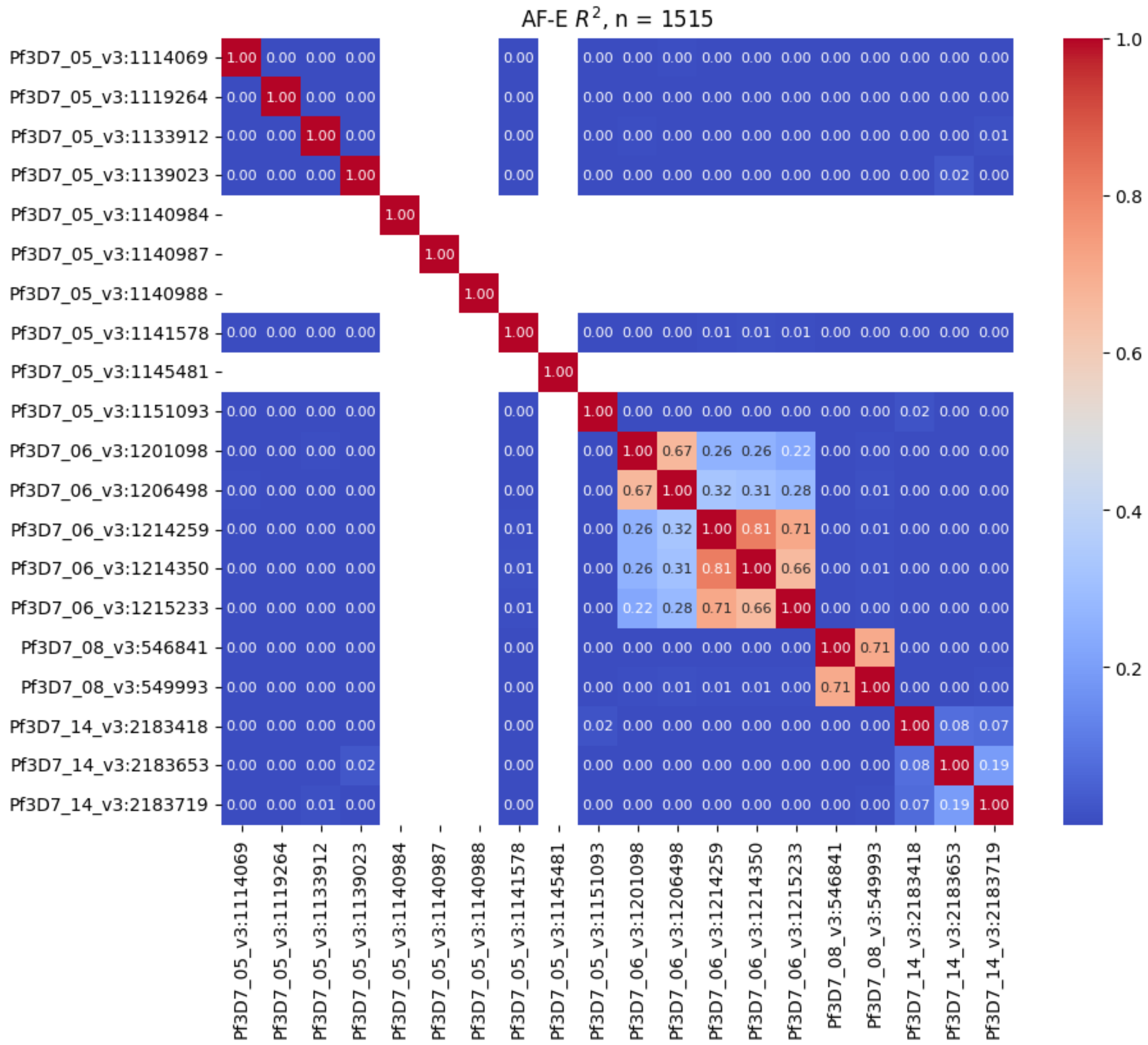


**Supplementary Figure S5**. Linkage disequilibrium ($R^2$) for the 20 most differentiated SNPs between Madagascar and East Africa. Shown here are the $R^2$ values for the East African population. Missing values indicate the SNP was not present in the population.

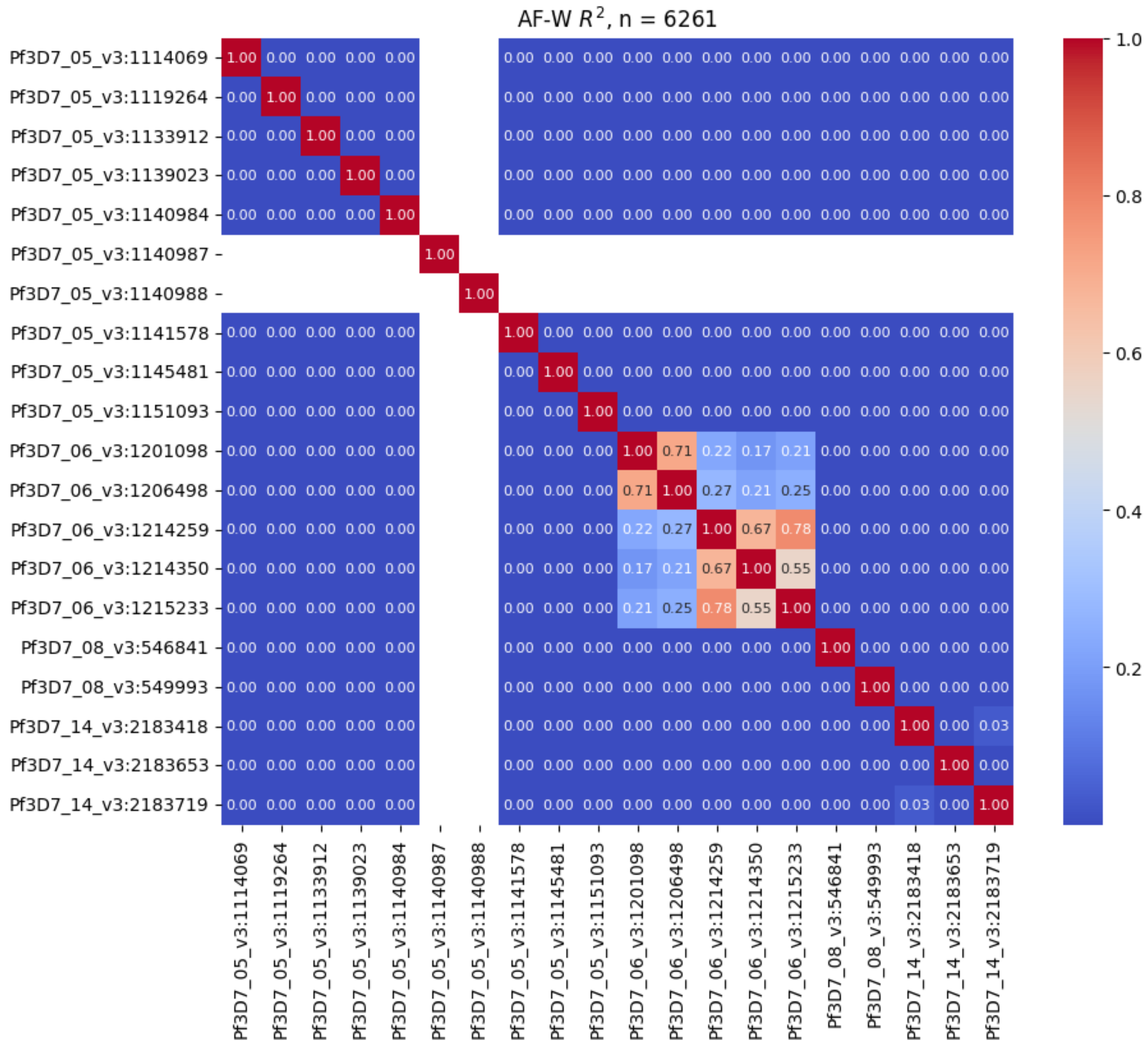

**Supplementary Figure S6**. Linkage disequilibrium ($R^2$) for the 20 most differentiated SNPs between Madagascar and East Africa. Shown here are the $R^2$ values for the West African population. Missing values indicate the SNP was not present in the population.

**Supplementary Table S1.** *dhfr* haplotype frequencies by population**.** Haplotype gives the amino acid changes with respect to the 3D7 reference genome, which here is assumed to be the wild type (WT) sequence. Table shows numbers of samples with a homozygous call with each haplotype. Column headings show populations as defined in Pf7: AF-W=Africa-West, AF-C=Africa-Central, AF-NE=Africa-Northeast, AF-E=Africa East. See Pf7 manuscript for further details [1]

| Haplotype | Number of samples | | | | |
|---|---|---|---|---|---|
| | AF-W | AF-C | AF-NE | AF-E | Total |
| **N51I/C59R/S108N** | 3,905 | 373 | 100 | 1,085 | 5,463 |
| **WT** | 661 | 2 | 3 | 47 | 713 |
| **C59R/S108N** | 260 | 3 | 1 | 75 | 339 |
| **N51I/S108N** | 52 | 59 | 45 | 99 | 255 |
| **S108N** | 17 | 2 | 2 | 4 | 25 |
| **N51I** | 0 | 0 | 0 | 2 | 2 |
| **N51I/C59R/S108N/I164L** | 0 | 0 | 0 | 2 | 2 |
| **N157K** | 2 | 0 | 0 | 0 | 2 |
| **C59R** | 1 | 0 | 0 | 0 | 1 |
| **N51I/S108N/I164L** | 0 | 0 | 1 | 0 | 1 |
| **K256R** | 1 | 0 | 0 | 0 | 1 |
| **N51I/C59R/S108N/D414N** | 1 | 0 | 0 | 0 | 1 |
| **N51I/C59R/S108N/D425N** | 1 | 0 | 0 | 0 | 1 |
| **M337K** | 1 | 0 | 0 | 0 | 1 |
| **N51I/C59R/P93S/S108N** | 1 | 0 | 0 | 0 | 1 |
| **T130N** | 1 | 0 | 0 | 0 | 1 |
| **S306F** | 0 | 0 | 0 | 1 | 1 |
| **N90Y** | 1 | 0 | 0 | 0 | 1 |
| **N51I/S108N/S306F** | 0 | 0 | 0 | 1 | 1 |
| **Total** | 4,905 | 439 | 152 | 1,316 | 6,812 |

**Supplementary Table S2.** Fisher's exact test for a change in resistance allele frequency between the first and final years sampled for *dhfr* loci in four African countries. P-values are adjusted for multiple testing with a Benjamini and Hochberg False Discovery Rate correction [2]. Significance is determined by an adjusted p-value < 0.05: *** for p-value < 0.001, ** for p-value 0.001 - 0.01, * for p-value 0.01 - 0.05, and NS (not significant) for p-value ≥ 0.05.

| Country | Locus | First Year | Final Year | Adjusted p-value | Significance |
|---|---|---|---|---|---|
| Gambia | dhfr_108 | 1984 | 2017 | 1.14E-34 | *** |
| | dhfr_164 | 1984 | 2017 | 1 | NS |
| | dhfr_51 | 1984 | 2017 | 4.86E-43 | *** |
| | dhfr_59 | 1984 | 2017 | 2.90E-38 | *** |
| Ghana | dhfr_108 | 2009 | 2018 | 0.0001 | *** |
| | dhfr_164 | 2009 | 2018 | 1 | NS |
| | dhfr_51 | 2009 | 2018 | 1.21E-05 | *** |
| | dhfr_59 | 2009 | 2018 | 0.0023 | ** |
| Kenya | dhfr_108 | 1995 | 2014 | 7.09E-10 | *** |
| | dhfr_164 | 1995 | 2014 | 1 | NS |
| | dhfr_51 | 1995 | 2014 | 1.90E-18 | *** |
| | dhfr_59 | 1995 | 2014 | 2.43E-10 | *** |
| Mali | dhfr_108 | 2007 | 2017 | 0.0021 | ** |
| | dhfr_164 | 2007 | 2017 | 1 | NS |
| | dhfr_51 | 2007 | 2017 | 0.0015 | ** |
| | dhfr_59 | 2007 | 2017 | 0.0009 | *** |

**Supplementary Table S3.** *dhps* haplotype frequencies by population**.** Haplotype gives the amino acid changes with respect to what is considered to be the wild type (WT) haplotype. Note that in this case, the 3D7 reference genome is not considered to be wild type, as the ancestral allele at amino acid 437 is thought to be alanine (A) where 3D7 has glycine (G). Table shows numbers of samples with a homozygous call with each haplotype. Column headings show populations as defined in Pf7: AF-W=Africa-West, AF-C=Africa-Central, AF-NE=Africa-Northeast, AF-E=Africa East. See Pf7 manuscript for further details [1].

| Haplotype | Number of samples | | | | |
|---|---|---|---|---|---|
| | AF-W | AF-C | AF-NE | AF-E | Total |
| **A437G** | 1,770 | 346 | 2 | 10 | 2,128 |
| **A437G/K540E** | 34 | 23 | 91 | 907 | 1,055 |
| **S436A/A437G** | 943 | 19 | 0 | 1 | 963 |
| **S436A** | 636 | 5 | 3 | 21 | 665 |
| **WT** | 279 | 12 | 20 | 150 | 461 |
| **S436A/A437G/A613S** | 202 | 0 | 0 | 0 | 202 |
| **A437G/K540E/A581G** | 0 | 11 | 12 | 134 | 157 |
| **I431V/S436A/A437G/A581G/A613S** | 72 | 4 | 0 | 0 | 76 |
| **A437G/I484T** | 27 | 0 | 0 | 0 | 27 |
| **E189Q/S436A** | 24 | 0 | 0 | 0 | 24 |
| **I431V/S436A/A437G** | 20 | 0 | 0 | 0 | 20 |
| **S436F/A613S** | 16 | 0 | 0 | 1 | 17 |
| **S152I/A437G** | 16 | 0 | 0 | 0 | 16 |
| **S436C** | 16 | 0 | 0 | 0 | 16 |
| **S436Y/A613S** | 13 | 0 | 0 | 0 | 13 |
| **S436H/A437G/K540E** | 0 | 0 | 7 | 0 | 7 |
| **E189Q/A437G** | 7 | 0 | 0 | 0 | 7 |
| **D280N** | 7 | 0 | 0 | 0 | 7 |
| **A437G/A581G** | 0 | 0 | 0 | 4 | 4 |
| **I431V/S436A/A437G/A613S** | 4 | 0 | 0 | 0 | 4 |
| **A437G/A613S** | 4 | 0 | 0 | 0 | 4 |
| **S436A/A437G/A581G/A613S** | 3 | 0 | 0 | 0 | 3 |
| **R243K** | 3 | 0 | 0 | 0 | 3 |
| **S436A/A613S** | 2 | 0 | 0 | 0 | 2 |
| **V471L** | 2 | 0 | 0 | 0 | 2 |
| **L22F/S436A** | 2 | 0 | 0 | 0 | 2 |
| **R243S/S436A** | 2 | 0 | 0 | 0 | 2 |
| **N13K** | 1 | 0 | 0 | 0 | 1 |
| **R243S** | 1 | 0 | 0 | 0 | 1 |
| **S436A/A437G/K540E** | 0 | 0 | 0 | 1 | 1 |
| **R27K** | 1 | 0 | 0 | 0 | 1 |
| **R78H** | 1 | 0 | 0 | 0 | 1 |
| **S10F** | 0 | 0 | 0 | 1 | 1 |

| | | | | | |
|---|---|---|---|---|---|
| **S436H** | 0 | 0 | 0 | 1 | 1 |
| **L166M** | 0 | 0 | 0 | 1 | 1 |
| **S436A/A672G** | 1 | 0 | 0 | 0 | 1 |
| **L263I** | 1 | 0 | 0 | 0 | 1 |
| **S436A/A581G/A613S** | 1 | 0 | 0 | 0 | 1 |
| **G117E** | 1 | 0 | 0 | 0 | 1 |
| **E189Q/S436A/A437G** | 1 | 0 | 0 | 0 | 1 |
| **K106I/A437G/K540E** | 1 | 0 | 0 | 0 | 1 |
| **D105H** | 1 | 0 | 0 | 0 | 1 |
| **I277T/S436A/A437G** | 1 | 0 | 0 | 0 | 1 |
| **I277L** | 1 | 0 | 0 | 0 | 1 |
| **K540E** | 0 | 0 | 0 | 1 | 1 |
| **D311G/A437G** | 1 | 0 | 0 | 0 | 1 |
| **D250V** | 1 | 0 | 0 | 0 | 1 |
| **E189Q** | 1 | 0 | 0 | 0 | 1 |
| **C351Y/S436A/A437G** | 0 | 1 | 0 | 0 | 1 |
| **E71Q** | 0 | 0 | 0 | 1 | 1 |
| **A437G/I441M** | 1 | 0 | 0 | 0 | 1 |
| **Total** | 4,121 | 421 | 135 | 1,234 | 5,911 |

**Supplementary Table S4:** Fisher's exact test for a change in resistance allele frequency between the first and final years sampled for *dhps* loci in four African countries. P-values are adjusted for multiple testing with a Benjamini and Hochberg False Discovery Rate correction [2]. Significance is determined by an adjusted p-value < 0.05: *** for p-value < 0.001, ** for p-value 0.001 - 0.01, * for p-value 0.01 - 0.05, and NS (not significant) for p-value ≥ 0.05

| Country | Locus | First Year | Last Year | Adjusted p-value | Significance |
|---|---|---|---|---|---|
| Gambia | dhps_431 | 1984 | 2017 | 1 | NS |
| | dhps_436 | 1984 | 2017 | 0.0005 | *** |
| | dhps_437 | 1984 | 2017 | 1.16E-20 | *** |
| | dhps_540 | 1984 | 2017 | 1 | NS |
| | dhps_581 | 1984 | 2017 | 1 | NS |
| | dhps_613 | 1984 | 2017 | 1 | NS |
| Ghana | dhps_431 | 2009 | 2018 | 1 | NS |
| | dhps_436 | 2009 | 2018 | 1 | NS |
| | dhps_437 | 2009 | 2018 | 1 | NS |
| | dhps_540 | 2009 | 2018 | 1 | NS |
| | dhps_581 | 2009 | 2018 | 1 | NS |
| | dhps_613 | 2009 | 2018 | 0.5515 | NS |
| Kenya | dhps_431 | 1995 | 2014 | 1 | NS |
| | dhps_436 | 1995 | 2014 | 1 | NS |
| | dhps_437 | 1995 | 2014 | 6.83E-26 | *** |
| | dhps_540 | 1995 | 2014 | 1.56E-28 | *** |
| | dhps_581 | 1995 | 2014 | 1 | NS |
| | dhps_613 | 1995 | 2014 | 1 | NS |
| Mali | dhps_431 | 2007 | 2017 | 0.72 | NS |
| | dhps_436 | 2007 | 2017 | 0.72 | NS |
| | dhps_437 | 2007 | 2017 | 6.06E-05 | *** |
| | dhps_540 | 2007 | 2017 | 1 | NS |
| | dhps_581 | 2007 | 2017 | 0.72 | NS |
| | dhps_613 | 2007 | 2017 | 0.3812 | NS |

**Supplementary Table S5.** Single mutations proposed as markers for multi-gene haplotypes.

| Maker Name | Marker | Multi Mutant Haplotype |
|---|---|---|
| Triple | *dhfr* C59R | *dhfr* N51I / C59R / S108N |
| Quintuple | *dhfr* C59R and *dhps* K540E | *dhfr* N51I / C59R / S108N, *dhps* A437G / K540E |
| Sextuple | *dhps* A581G | *dhfr* N51I / C59R / S108N, *dhps* A437G / K540E / A581G |

## References


1. MalariaGEN *et al.* Pf7: an open dataset of Plasmodium falciparum genome variation in 20,000 worldwide samples. *Wellcome Open Res.* **8**, 22 (2023).
2. Benjamini, Y. & Hochberg, Y. Controlling the False Discovery Rate: A Practical and Powerful Approach to Multiple Testing. *J. R. Stat. Soc. Ser. B Methodol.* **57**, 289–300 (1995).
